# Supplementary material for: Can social support buffer the association between loneliness and hypertension? a cross-sectional study in rural China
Source: PLoS One. 2022 Feb 18;17(2):e0264086. doi: 10.1371/journal.pone.0264086 (PMC8856532; doi:10.1371/journal.pone.0264086)
Supplement: S1 File — (DOCX) [file pone.0264086.s001.docx]

**Supplementary.**

*Measurement of social capital*

We assessed social capital in terms of trust, reciprocity, and attachment to other community members with the following questions; “Generally speaking, do you trust people in your community?”, “Do people in your community try to be helpful to others?” and “How attached do you feel to your community?”, respectively using a five-point Likert scale (i.e., never, rarely, sometimes, most of the time, always). Means (standard deviations) were 3.5 (0.76), 3.7 (0.92), and 3.5 (0.88) respectively. Unadjusted correlations between loneliness score (1 – 5) and each social capital measure were calculated by a least-squares regression analysis.
