# Supplementary material for: Can social support buffer the association between loneliness and hypertension? a cross-sectional study in rural China
Source: PLoS One. 2022 Feb 18;17(2):e0264086. doi: 10.1371/journal.pone.0264086 (PMC8856532; doi:10.1371/journal.pone.0264086)
Supplement: S1 Appendix — (DOCX) [file pone.0264086.s002.docx]

Appendix. Characteristics of social support receiver/provider (n = 763).

|  | Instrumental social support | | | | |  | Emotional social support | | | |
| --- | --- | --- | --- | --- | --- | --- | --- | --- | --- | --- |
|  | None | Receipt only | Provision only | Receipt & provision | p |  | Low | Middle | High | p |
| Age | 59.7 [12.6] | 57.3 [13.6] | 59.6 [15.6] | 57.2 [13.3] | 0.12 |  | 62.7 [11.8] | 55.0 [13.0] | 56.7 [12/9] | <0.001 |
| Sex (male) | 230 (42.8) | 4 (14.8) | 6 (46.2) | 57 (30.7) | 0.001 |  | 147 (40.1) | 90 (35.2) | 60 (42.9) | 0.27 |
| BMI category |  |  |  |  |  |  |  |  |  |  |
| Underweight | 23 (4.3) | 2 (7.4) | 3 (23.1) | 7 (3.8) | 0.046 |  | 19 (5.2) | 9 (3.5) | 7 (5.0) | 0.77 |
| Normal | 239 (44.5) | 10 (37.0) | 8 (61.5) | 93 (50.0) |  |  | 170 (46.3) | 116 (45.3) | 64 (45.7) |  |
| Overweight | 219 (40.8) | 11 (40.7) | 2 (15.4) | 67 (36.0) |  |  | 140 (38.2) | 108 (42.2) | 51 (36.4) |  |
| Obese | 56 (10.4) | 4 (14.8) | 0 (0.0) | 19 (10.2) |  |  | 38 (10.4) | 23 (9.0) | 18 (12.9) |  |
| Marriage |  |  |  |  |  |  |  |  |  |  |
| Has a partner | 20 (3.7) | 2 (7.4) | 1 (7.7) | 6 (3.2) | 0.50 |  | 281 (76.6) | 215 (84.0) | 115 (82.1) | 0.12 |
| Not married | 422 (78.6) | 21 (77.8) | 10 (76.9) | 158 (85.0) |  |  | 14 (2.8) | 8 (3.1) | 7 (5.0) |  |
| Divorced or widowed | 95 (17.7) | 4 (14.8) | 2 (15.4) | 22 (11.8) |  |  | 72 (16.6) | 33 (12.9) | 18 (12.9) |  |
| Education |  |  |  |  |  |  |  |  |  |  |
| Illiterate | 199 (37.1) | 9 (33.3) | 7 (53.9) | 70 (37.6) | 0.056 |  | 185 (50.4) | 64 (25.0) | 36 (25.7) | <0.001 |
| Less than elementary school | 227 (42.3) | 13 (48.2) | 4 (30.8) | 59 (31.7) |  |  | 142 (38.7) | 108 (42.2) | 53 (37.9) |  |
| Junior high school or more | 111 (20.7) | 5 (18.5) | 2 (15.4) | 57 (30.7) |  |  | 40 (10.9) | 84 (32.8) | 51 (36.4) |  |
| Employment |  |  |  |  |  |  |  |  |  |  |
| Not currently employed | 145 (27.0) | 5 (18.5) | 2 (15.4) | 37 (19.9) | 0.19 |  | 98 (26.7) | 53 (20.7) | 38 (27.1) | <0.001 |
| Farming/fishing | 273 (50.8) | 15 (55.6) | 8 (61.5) | 120 (64.5) |  |  | 217 (59.1) | 127 (49.6) | 72 (51.4) |  |
| Self employed | 21 (3.9) | 3 (11.1) | 0 (0.0) | 7 (3.8) |  |  | 9 (2.5) | 16 (6.3) | 6 (4.3) |  |
| Formal employee | 9 (1.7) | 0 (0.0) | 0 (0.0) | 1 (0.5) |  |  | 0 (0.0) | 8 (3.1) | 2 (1.4) |  |
| Part-time job with heavy physical activity | 33 (6.2) | 2 (7.4) | 0 (0.0) | 8 (4.3) |  |  | 14 (3.8) | 23 (9.0) | 6 (4.3) |  |
| Part-time job with low-moderate physical activity | 49 (9.1) | 2 (7.4) | 3 (23.1) | 13 (7.0) |  |  | 24 (6.5) | 27 (10.6) | 16 (11.4) |  |
| Others | 7 (1.3) | 0 (0.0) | 0 (0.0) | 0 (0.0) |  |  | 5 (1.4) | 2 (0.8) | 0 (0.0) |  |
| Self-rated household income |  |  |  |  |  |  |  |  |  |  |
| Low | 246 (45.8) | 14 (51.9) | 8 (61.5) | 67 (36.0) | 0.20 |  | 183 (49.9) | 108 (42.2) | 44 (31.4) | <0.001 |
| Middle | 218 (40.6) | 9 (33.3) | 4 (30.8) | 93 (50.0) |  |  | 157 (42.8) | 105 (41.0) | 62 (44.3) |  |
| High | 73 (13.6) | 4 (14.8) | 1 (7.7) | 26 (14.0) |  |  | 27 (7.4) | 43 (16.8) | 34 (24.3) |  |
| Alcohol consumption |  |  |  |  |  |  |  |  |  |  |
| Does not drink | 384 (71.5) | 23 (85.2) | 7 (53.9) | 150 (80.7) | <0.001 |  | 282 (76.8) | 186 (72.7) | 96 (68.6) | 0.53 |
| 1 or 2 days a week | 40 (7.5) | 2 (7.4) | 3 (23.1) | 3 (1.6) |  |  | 19 (5.2) | 19 (7.4) | 10 (7.1) |  |
| 3 to 6 days a week | 9 (1.7) | 0 (0.0) | 2 (15.4) | 2 (1.1) |  |  | 6 (1.6) | 4 (1.6) | 3 (2.1) |  |
| Every day | 54 (10.1) | 1 (3.7) | 1 (7.7) | 15 (8.1) |  |  | 35 (9.5) | 20 (7.8) | 16 (11.4) |  |
| Every day (Heavy) | 50 (9.3) | 1 (3.7) | 0 (0.0) | 16 (8.6) |  |  | 25 (6.8) | 27 (10.6) | 15 (10.7) |  |
| Smoking |  |  |  |  |  |  |  |  |  |  |
| Never smoked | 415 (77.3) | 27 (100.0) | 8 (61.5) | 155 (83.3) | 0.035 |  | 297 (80.9) | 205 (80.1) | 103 (73.6) | 0.036 |
| Has stopped smoking | 41 (7.6) | 0 (0.0) | 1 (7.7) | 11 (5.9) |  |  | 31 (8.5) | 11 (4.3) | 11 (7.9) |  |
| Currently smoke | 81 (15.1) | 0 (0.0) | 4 (30.8) | 20 (10.8) |  |  | 39 (10.6) | 40 (15.6) | 26 (18.6) |  |
| Physical activity |  |  |  |  |  |  |  |  |  |  |
| Low | 187 (34.8) | 7 (25.9) | 4 (30.8) | 43 (23.1) | 0.017 |  | 149 (40.6) | 56 (21.9) | 36 (25.7) | <0.001 |
| Middle | 148 (27.6) | 4 (14.8) | 5 (38.5) | 67 (36.0) |  |  | 84 (22.9) | 96 (37.5) | 44 (31.4) |  |
| High | 202 (37.6) | 16 (59.3) | 4 (30.8) | 76 (40.9) |  |  | 134 (36.5) | 104 (40.6) | 60 (42.9) |  |

BMI; body mass index. Mean and standard deviation for continuous variables and number of participants and the percentage for categorical variables are shown (mean [SD]/n(%)). *P*-values for Chi-squared test for categorical variables and the analysis of variance for continuous variable are shown.
