# Supplementary material for: Can social support buffer the association between loneliness and hypertension? a cross-sectional study in rural China
Source: PLoS One. 2022 Feb 18;17(2):e0264086. doi: 10.1371/journal.pone.0264086 (PMC8856532; doi:10.1371/journal.pone.0264086)
Supplement: Attachment — Submitted filename: Response to Reviewers.docx [file pone.0264086.s003.docx]

**Response letter**

PONE-D-21-28413

**Reviewer #1:** 

Thank you very much for your careful review and constructive suggestions. Please see our detailed responses to your comments on our manuscript.

**Introduction**

1. Line 77–: I understand that the aim of this study was to evaluate 1) the association of the sense of loneliness and high blood pressure in rural China and 2) the role of social relationships in the association. Thus, it seems to me that “the specific mechanisms linking each type of social support and health outcomes remain unclear” is not relevant.

Answer 1: We thank the reviewer for pointing this out. We agree with the argument, and we have deleted the sentence as per the suggestion.

2. Line 82 & 83: I am confused with your usage of “positive” and “negative” in the last sentence of Introduction; I would replace “negative” with “positive.”

Answer 2: We apologize for our poor description and greatly appreciate the reviewer for highlighting this issue. We have replaced the word as the reviewer suggested.

[P.4 L.79] The authors hypothesized that (1) loneliness is positively associated with hypertension; and (2) social support from neighbors would mitigate (buffer) the association between loneliness and hypertension (i.e., high social support would protect against the positive association of loneliness on hypertension).

**Materials and methods**

1. Line 89–: You mentioned “the basis of average population size and level of economic development.” I think you should be more specific and explain the criteria you adopted.

Answer 1: We appreciate the suggestion. Following this comment, we have added detailed information about the situation in the communities as follows:

[P.4 L.88] Median household income based on the responses from 161 household heads (i.e., 11,000 RMB, interquartile range: 4800 – 24000 RMB) was comparable to figure reported for rural communities in the area as of 2014 (i.e., 11,252 RMB) (1 RMB = 0.16 USD as of 2015) [21].

2. Line 95–: I am wondering how the difference of the excluded samples and the included ones could impact on the estimates. Could you mention the possible impact in Discussion?

Answer 2: We thank the reviewer for this comment. The exclusion of those with missing values might have biased the observed associations. For example, it is possible that those with missing values might have had co-morbid health-related problems which prevented them from communicating with others or engaging in social participation. While multiple imputation is often used to account for missingness, we have decided not to conduct multiple imputation on this occasion as the missing at random (MAR) assumption seems to be seriously violated. As a response to this comment, the following sentences were added in the Limitation section.

[P.17 L.349] Lastly, the exclusion of those with missing values might have biased the observed associations toward the null, e.g., those with missing values might have had health-related problems which prevented them from engaging in social interactions.

3. Line 116–: I am wondering why only “receiving emotional support” was evaluated and “providing” emotional support was not. Furthermore, how could you claim that the stated number of friends represented just the “reception” of emotional support, instead of reciprocal friendship? I am also concerned about the validity of the three measures of social support.

Answer 3: We greatly appreciate the reviewer for raising this critical issue. This reviewer’s comment allowed us to realize that we failed to accurately label the variable. We collected information on the number of friends (“the number of close friends one can turn to for immediate help when the respondent is in trouble”, to be exact) to assess the structural aspect of one’s social network that are characterized by reciprocal emotional support. As a response to this comment, we have termed the variable “reciprocal emotional support”.

In addition, to make the instrumental support variable correspond to the item on emotional support, we have decided to combine provision and receipt of instrumental support into one categorical variable termed “reciprocal instrumental support”. This variable has four categories, i.e., (1) only receiving instrumental support, (2) only providing instrumental support, (3) both receiving and providing instrumental support, and (4) neither receiving nor providing instrumental support. Table 1, 2, and Appendix were all revised accordingly.

We have also mentioned that these social support variables have not been validated in the Limitation section.

[Abstract] *Methods.* Social support was measured as the reciprocal instrumental social support from/to neighbors and the reciprocal emotional support (i.e., the number of close friends that the respondent could turn to for help immediately when they are in trouble).

[Abstract] *Results.* Analysis revealed that those who were lonely had a higher prevalence ratio for hypertension (prevalence ratio = 1.12, 95% confidence interval 0.99 – 1.26) compared to those who reported not being lonely. There was an interaction between social support and loneliness in relation to hypertension. Specifically, contrary to the stress buffering theory, the positive association between loneliness and hypertension was more pronounced among those who reported higher social support compared to those who reported lower support (*p* for interaction <0.001 for instrumental support).

[P.5 L.113] Social support was measured in two dimensions: reciprocal instrumental support and reciprocal emotional support. Reciprocal instrumental support was measured as the reported frequency of exchanging (receiving or providing) various commodities (e.g., food and medicines) between neighbors. Data on the frequency of receiving and providing instrumental support were separately obtained to create four categories (i.e., neither receiving or providing instrumental support; only receiving instrumental support; only providing instrumental support; both receiving and providing instrumental support). Reciprocal emotional support was assessed as the number of close friends that the respondent could turn to for help immediately when they are in trouble, which was then categorized into three groups (none; 1 to 5; 6 or more) since the number of friends ranged from 0 – 35, and 48% of the participants answered they have no friend.

[P.16 L.300] We used the number of close friends to whom people could turn to for help when they are in trouble as an indicator of expected emotional support between close friends

[P.17 L.340] Fourth, we did not ask receipt and provision of emotional support separately.

[P.17 L.344] Furthermore, our social support measures have not been validated against existing instruments.

Table 1. Basic characteristics of the study participants (n = 763)

|  | **Total** | **Loneliness** | |
| --- | --- | --- | --- |
|  | **(n = 763)** | **Yes (n = 295)** | **No (n = 468)** |
| Age (in years) | 59.0 [12.9] | 61.7 [12.0] | 57.4 [13.2] |
| Sex (Male) | 297 (38.9) | 106 (35.9) | 191 (40.8) |
| BMI |  |  |  |
| Underweight | 35 (4.6) | 12 (4.1) | 23 (4.9) |
| Normal | 350 (45.9) | 130 (44.1) | 220 (47.0) |
| Overweight | 299 (39.2) | 120 (40.7) | 179 (38.3) |
| Obese | 79 (10.4) | 33 (11.2) | 46 (9.8) |
| Marital status |  |  |  |
| Has a partner | 611 (80.1) | 213 (72.2) | 398 (85.0) |
| Not married | 29 (3.8) | 10 (3.4) | 19 (4.1) |
| Divorced or widowed | 123 (16.1) | 72 (24.4) | 51 (10.9) |
| Education |  |  |  |
| Illiterate | 285 (37.4) | 137 (46.4) | 148 (31.6) |
| Less than elementary school | 303 (39.7) | 117 (39.7) | 186 (39.7) |
| Junior high school or more | 175 (22.9) | 41 (13.9) | 134 (28.6) |
| Employment |  |  |  |
| Not currently employed | 189 (24.8) | 84 (28.5) | 105 (22.4) |
| Farming/fishing | 416 (54.5) | 169 (57.3) | 247 (52.8) |
| Self-employment | 31 (4.1) | 12 (4.1) | 19 (4.1) |
| Formal employee | 10 (1.3) | 2 (0.7) | 8 (1.7) |
| Part-time job with heavy physical activity | 43 (5.6) | 9 (3.1) | 34 (7.3) |
| Part-time job with low-moderate physical activity | 67 (8.8) | 16 (5.4) | 51 (10.9) |
| Others | 7 (0.9) | 3 (1.0) | 4 (0.9) |
| Household income |  |  |  |
| Low | 335 (43.9) | 156 (52.9) | 179 (38.3) |
| Middle | 324 (42.5) | 99 (33.6) | 225 (48.1) |
| High | 104 (13.6) | 40 (13.6) | 64 (13.7) |
| Alcohol consumption |  |  |  |
| Does not drink | 564 (73.9) | 232 (78.6) | 332 (70.9) |
| 1 or 2 days a week | 48 (6.3) | 20 (6.8) | 28 (6.0) |
| 3 to 6 days a week | 13 (1.7) | 4 (1.4) | 9 (1.9) |
| Every day | 71 (9.3) | 21 (7.1) | 50 (10.7) |
| Every day (heavy) | 67 (8.8) | 18 (6.1) | 49 (10.5) |
| Smoking |  |  |  |
| Never smoked | 605 (79.3) | 237 (80.3) | 368 (78.6) |
| Has stopped smoking | 53 (7.0) | 20 (6.8) | 33 (7.1) |
| Currently smoke | 105 (13.8) | 38 (12.9) | 67 (14.3) |
| Physical activity |  |  |  |
| Low | 241 (31.6) | 112 (38.0) | 129 (27.6) |
| Middle | 224 (29.4) | 79 (26.8) | 145 (31.0) |
| High | 298 (39.1) | 104 (35.3) | 194 (41.5) |
| Instrumental support |  |  |  |
| None | 537 (70.4) | 231 (78.3) | 306 (65.4) |
| Receipt only | 27 (3.5) | 11 (3.7) | 16 (3.4) |
| Provision only | 13 (1.7) | 4 (1.4) | 9 (1.9) |
| Both receipt and provision | 186 (24.4) | 49 (16.6) | 137 (29.3) |
| Emotional support (Number of friends) |  |  |  |
| None | 367 (48.1) | 162 (54.9) | 205 (43.8) |
| 1 to 5 | 256 (33.6) | 89 (30.2) | 167 (35.7) |
| 6 or more | 140 (18.4) | 44 (14.9) | 96 (20.5) |

BMI; body mass index. Mean [standard deviation] or n (%) are shown.

Table 2. Results of Poisson regression model with robust variance estimator examining the association between loneliness, social support and hypertension among rural community dwellers in Fujian Province, China (n = 763)

|  | **Model 1** | **Model 2** | **Model 3** |
| --- | --- | --- | --- |
| Loneliness (ref. Low) | 1.12 (0.99, 1.26) | 1.04 (0.89, 1.22) | 1.05 (0.91, 1.20) |
| Instrumental support (ref. None) |  |  |  |
| Receipt only |  | 1.08 (0.75, 1.57) | 1.07 (0.75, 1.53) |
| Provision only |  | 1.07 (0.59, 1.95) | 1.09 (0.69, 1.73) |
| Both receipt and provision |  | 0.99 (0.83, 1.19) | 1.14 (0.98, 1.33) |
| Emotional support (ref. Low) |  |  |  |
| Middle |  | 0.91 (0.80, 1.05) | 0.88 (0.75. 1.03) |
| High |  | 0.83 (0.73, 0.95) | 0.72 (0.60, 0.87) |
| Loneliness x Instrumental support (ref. None) |  |  |  |
| Receipt only |  | 0.95 (0.58, 1.58) |  |
| Provision only |  | 1.08 (0.32, 3.57) |  |
| Both receipt and provision |  | 1.49 (1.20, 1.87) |  |
| Loneliness x Emotional support (ref. Low) |  |  |  |
| Middle |  |  | 1.09 (0.92, 1.28) |
| High |  |  | 1.44 (0.90, 2.33) |

Values are prevalence ratios and 95% confidence intervals. Covariates included age, age-squared, sex, body mass index category, marital status, educational attainment, employment status, household income, alcohol consumption, smoking and physical activity. A random effects model was used to account for multiple individuals in each community.

Appendix. Characteristics of social support receiver/provider (n = 763).

|  | Instrumental social support | | | | |  | Emotional social support | | | |
| --- | --- | --- | --- | --- | --- | --- | --- | --- | --- | --- |
|  | None | Receipt only | Provision only | Receipt & provision | p |  | Low | Middle | High | p |
| Age | 59.7 [12.6] | 57.3 [13.6] | 59.6 [15.6] | 57.2 [13.3] | 0.12 |  | 62.7 [11.8] | 55.0 [13.0] | 56.7 [12/9] | <0.001 |
| Sex (male) | 230 (42.8) | 4 (14.8) | 6 (46.2) | 57 (30.7) | 0.001 |  | 147 (40.1) | 90 (35.2) | 60 (42.9) | 0.27 |
| BMI category |  |  |  |  |  |  |  |  |  |  |
| Underweight | 23 (4.3) | 2 (7.4) | 3 (23.1) | 7 (3.8) | 0.046 |  | 19 (5.2) | 9 (3.5) | 7 (5.0) | 0.77 |
| Normal | 239 (44.5) | 10 (37.0) | 8 (61.5) | 93 (50.0) |  |  | 170 (46.3) | 116 (45.3) | 64 (45.7) |  |
| Overweight | 219 (40.8) | 11 (40.7) | 2 (15.4) | 67 (36.0) |  |  | 140 (38.2) | 108 (42.2) | 51 (36.4) |  |
| Obese | 56 (10.4) | 4 (14.8) | 0 (0.0) | 19 (10.2) |  |  | 38 (10.4) | 23 (9.0) | 18 (12.9) |  |
| Marriage |  |  |  |  |  |  |  |  |  |  |
| Has a partner | 20 (3.7) | 2 (7.4) | 1 (7.7) | 6 (3.2) | 0.50 |  | 281 (76.6) | 215 (84.0) | 115 (82.1) | 0.12 |
| Not married | 422 (78.6) | 21 (77.8) | 10 (76.9) | 158 (85.0) |  |  | 14 (2.8) | 8 (3.1) | 7 (5.0) |  |
| Divorced or widowed | 95 (17.7) | 4 (14.8) | 2 (15.4) | 22 (11.8) |  |  | 72 (16.6) | 33 (12.9) | 18 (12.9) |  |
| Education |  |  |  |  |  |  |  |  |  |  |
| Illiterate | 199 (37.1) | 9 (33.3) | 7 (53.9) | 70 (37.6) | 0.056 |  | 185 (50.4) | 64 (25.0) | 36 (25.7) | <0.001 |
| Less than elementary school | 227 (42.3) | 13 (48.2) | 4 (30.8) | 59 (31.7) |  |  | 142 (38.7) | 108 (42.2) | 53 (37.9) |  |
| Junior high school or more | 111 (20.7) | 5 (18.5) | 2 (15.4) | 57 (30.7) |  |  | 40 (10.9) | 84 (32.8) | 51 (36.4) |  |
| Employment |  |  |  |  |  |  |  |  |  |  |
| Not currently employed | 145 (27.0) | 5 (18.5) | 2 (15.4) | 37 (19.9) | 0.19 |  | 98 (26.7) | 53 (20.7) | 38 (27.1) | <0.001 |
| Farming/fishing | 273 (50.8) | 15 (55.6) | 8 (61.5) | 120 (64.5) |  |  | 217 (59.1) | 127 (49.6) | 72 (51.4) |  |
| Self employed | 21 (3.9) | 3 (11.1) | 0 (0.0) | 7 (3.8) |  |  | 9 (2.5) | 16 (6.3) | 6 (4.3) |  |
| Formal employee | 9 (1.7) | 0 (0.0) | 0 (0.0) | 1 (0.5) |  |  | 0 (0.0) | 8 (3.1) | 2 (1.4) |  |
| Part-time job with heavy physical activity | 33 (6.2) | 2 (7.4) | 0 (0.0) | 8 (4.3) |  |  | 14 (3.8) | 23 (9.0) | 6 (4.3) |  |
| Part-time job with low-moderate physical activity | 49 (9.1) | 2 (7.4) | 3 (23.1) | 13 (7.0) |  |  | 24 (6.5) | 27 (10.6) | 16 (11.4) |  |
| Others | 7 (1.3) | 0 (0.0) | 0 (0.0) | 0 (0.0) |  |  | 5 (1.4) | 2 (0.8) | 0 (0.0) |  |
| Self-rated household income |  |  |  |  |  |  |  |  |  |  |
| Low | 246 (45.8) | 14 (51.9) | 8 (61.5) | 67 (36.0) | 0.20 |  | 183 (49.9) | 108 (42.2) | 44 (31.4) | <0.001 |
| Middle | 218 (40.6) | 9 (33.3) | 4 (30.8) | 93 (50.0) |  |  | 157 (42.8) | 105 (41.0) | 62 (44.3) |  |
| High | 73 (13.6) | 4 (14.8) | 1 (7.7) | 26 (14.0) |  |  | 27 (7.4) | 43 (16.8) | 34 (24.3) |  |
| Alcohol consumption |  |  |  |  |  |  |  |  |  |  |
| Does not drink | 384 (71.5) | 23 (85.2) | 7 (53.9) | 150 (80.7) | <0.001 |  | 282 (76.8) | 186 (72.7) | 96 (68.6) | 0.53 |
| 1 or 2 days a week | 40 (7.5) | 2 (7.4) | 3 (23.1) | 3 (1.6) |  |  | 19 (5.2) | 19 (7.4) | 10 (7.1) |  |
| 3 to 6 days a week | 9 (1.7) | 0 (0.0) | 2 (15.4) | 2 (1.1) |  |  | 6 (1.6) | 4 (1.6) | 3 (2.1) |  |
| Every day | 54 (10.1) | 1 (3.7) | 1 (7.7) | 15 (8.1) |  |  | 35 (9.5) | 20 (7.8) | 16 (11.4) |  |
| Every day (Heavy) | 50 (9.3) | 1 (3.7) | 0 (0.0) | 16 (8.6) |  |  | 25 (6.8) | 27 (10.6) | 15 (10.7) |  |
| Smoking |  |  |  |  |  |  |  |  |  |  |
| Never smoked | 415 (77.3) | 27 (100.0) | 8 (61.5) | 155 (83.3) | 0.035 |  | 297 (80.9) | 205 (80.1) | 103 (73.6) | 0.036 |
| Has stopped smoking | 41 (7.6) | 0 (0.0) | 1 (7.7) | 11 (5.9) |  |  | 31 (8.5) | 11 (4.3) | 11 (7.9) |  |
| Currently smoke | 81 (15.1) | 0 (0.0) | 4 (30.8) | 20 (10.8) |  |  | 39 (10.6) | 40 (15.6) | 26 (18.6) |  |
| Physical activity |  |  |  |  |  |  |  |  |  |  |
| Low | 187 (34.8) | 7 (25.9) | 4 (30.8) | 43 (23.1) | 0.017 |  | 149 (40.6) | 56 (21.9) | 36 (25.7) | <0.001 |
| Middle | 148 (27.6) | 4 (14.8) | 5 (38.5) | 67 (36.0) |  |  | 84 (22.9) | 96 (37.5) | 44 (31.4) |  |
| High | 202 (37.6) | 16 (59.3) | 4 (30.8) | 76 (40.9) |  |  | 134 (36.5) | 104 (40.6) | 60 (42.9) |  |

BMI; body mass index. Mean and standard deviation for continuous variables and number of participants and the percentage for categorical variables are shown (mean [SD]/n(%)). *P*-values for Chi-squared test for categorical variables and the analysis of variance for continuous variable are shown.

4. Line 127–: I think income and employment status were important confounders when considering the association of the sense of loneliness and health. Could you include these variables in this study? If you do not have the information, in what direction should the omitted-variable bias be?

Answer 4: Thank you for pointing out the important issue. We have added self-reported household income and employment status as covariates and updated the Methods and Tables accordingly. We did not see material changes in the study results when we included these variables in the models. Given that the information on income was not available for 2 participants, the total sample size became 763.

[P.5 L.128] employment status (not currently employed; farming/fishing; self-employed; formal employee; part-time job with heavy physical activity (e.g., construction workers), part-time job with low-moderate physical activity (e.g., office workers); others), and household income, which was self-reported on a 10-point Likert scale and categorized into tertiles (low; middle; high).

[P.6 L.152] Covariates included age, age-squared, sex, body mass index, marital status, educational attainment, employment, household income, alcohol consumption, smoking, and physical activity.

5. Line 138–: How about introducing community fixed-effects to account for time-invariant community specific characteristics?

Answer 5: We followed the reviewer’s question and ran the analysis with community fixed effects. The effect size for the association between loneliness and hypertension became smaller but trended in the same direction (PR = 1.10, 95% CI 0.96, 1.26). As a response to this comment, we have added the explanation in the Methods.

[P.6 L.150] A random effect model was chosen, given the large variations in the levels of urbanization across communities (the proportion of those engaging in farming/fishing: 53.2 – 80%) and population size (500 – 1220).

[P.7 L.164] Finally, the analysis with community fixed effects rather than random effects was conducted.

[P.13 L.234] When we ran the analysis with community fixed effects rather than random effects, the effect size for the association between loneliness and hypertension became smaller but trended in the same direction (PR = 1.10, 95% CI 0.96, 1.26).

6. Line 148–: I think SBP and DBP were used without making it clear what they stood for. Could you be specific?

Answer 6: Thanks for pointing this out. I have added the following sentence in the Methods.

[P.4 L.97] Systolic and diastolic blood pressure (SBP and DBP) were measured

**Results**

1. Table 1: I am wondering why Table 1 was presented according to the age group. I would replace this table by the table in Appendix. Furthermore, what “[]” and “()” indicated should be clear in this table.

Answer 1: Following the suggestion, we have amended Table 1 as shown below. I made a footnote ‘Mean [standard deviation] or n (%) are shown.’

Table 1. Basic characteristics of the study participants (n = 763)

|  | **Total** | **Loneliness** | |
| --- | --- | --- | --- |
|  | **(n = 763)** | **Yes (n = 295)** | **No (n = 468)** |
| Age (in years) | 59.0 [12.9] | 61.7 [12.0] | 57.4 [13.2] |
| Sex (Male) | 297 (38.9) | 106 (35.9) | 191 (40.8) |
| BMI |  |  |  |
| Underweight | 35 (4.6) | 12 (4.1) | 23 (4.9) |
| Normal | 350 (45.9) | 130 (44.1) | 220 (47.0) |
| Overweight | 299 (39.2) | 120 (40.7) | 179 (38.3) |
| Obese | 79 (10.4) | 33 (11.2) | 46 (9.8) |
| Marital status |  |  |  |
| Has a partner | 611 (80.1) | 213 (72.2) | 398 (85.0) |
| Not married | 29 (3.8) | 10 (3.4) | 19 (4.1) |
| Divorced or widowed | 123 (16.1) | 72 (24.4) | 51 (10.9) |
| Education |  |  |  |
| Illiterate | 285 (37.4) | 137 (46.4) | 148 (31.6) |
| Less than elementary school | 303 (39.7) | 117 (39.7) | 186 (39.7) |
| Junior high school or more | 175 (22.9) | 41 (13.9) | 134 (28.6) |
| Employment |  |  |  |
| Not currently employed | 189 (24.8) | 84 (28.5) | 105 (22.4) |
| Farming/fishing | 416 (54.5) | 169 (57.3) | 247 (52.8) |
| Self-employment | 31 (4.1) | 12 (4.1) | 19 (4.1) |
| Formal employee | 10 (1.3) | 2 (0.7) | 8 (1.7) |
| Part-time job with heavy physical activity | 43 (5.6) | 9 (3.1) | 34 (7.3) |
| Part-time job with low-moderate physical activity | 67 (8.8) | 16 (5.4) | 51 (10.9) |
| Others | 7 (0.9) | 3 (1.0) | 4 (0.9) |
| Household income |  |  |  |
| Low | 335 (43.9) | 156 (52.9) | 179 (38.3) |
| Middle | 324 (42.5) | 99 (33.6) | 225 (48.1) |
| High | 104 (13.6) | 40 (13.6) | 64 (13.7) |
| Alcohol consumption |  |  |  |
| Does not drink | 564 (73.9) | 232 (78.6) | 332 (70.9) |
| 1 or 2 days a week | 48 (6.3) | 20 (6.8) | 28 (6.0) |
| 3 to 6 days a week | 13 (1.7) | 4 (1.4) | 9 (1.9) |
| Every day | 71 (9.3) | 21 (7.1) | 50 (10.7) |
| Every day (heavy) | 67 (8.8) | 18 (6.1) | 49 (10.5) |
| Smoking |  |  |  |
| Never smoked | 605 (79.3) | 237 (80.3) | 368 (78.6) |
| Has stopped smoking | 53 (7.0) | 20 (6.8) | 33 (7.1) |
| Currently smoke | 105 (13.8) | 38 (12.9) | 67 (14.3) |
| Physical activity |  |  |  |
| Low | 241 (31.6) | 112 (38.0) | 129 (27.6) |
| Middle | 224 (29.4) | 79 (26.8) | 145 (31.0) |
| High | 298 (39.1) | 104 (35.3) | 194 (41.5) |
| Instrumental support |  |  |  |
| None | 537 (70.4) | 231 (78.3) | 306 (65.4) |
| Receipt only | 27 (3.5) | 11 (3.7) | 16 (3.4) |
| Provision only | 13 (1.7) | 4 (1.4) | 9 (1.9) |
| Both receipt and provision | 186 (24.4) | 49 (16.6) | 137 (29.3) |
| Emotional support (Number of friends) |  |  |  |
| None | 367 (48.1) | 162 (54.9) | 205 (43.8) |
| 1 to 5 | 256 (33.6) | 89 (30.2) | 167 (35.7) |
| 6 or more | 140 (18.4) | 44 (14.9) | 96 (20.5) |

Mean [standard deviation] or n (%) are shown.

2. Line 178–: Why were the mean blood pressure presented here? This information should rather be placed in “Characteristics of study participants.”

Answer 2. Following the suggestion, we have moved the sentence to Characteristics of study participants.

[P.8 L.182] The mean SBP and DBP was 140.9 and 82.6 mmHg among those who felt lonely, while it was 133.2 and 79.6 mmHg among those who did not feel lonely.

3. Line 182: “Fig 1” should be spelled out as “Figure 1.”

Answer 3. We appreciate this comment. We spelled it as per the author guideline.

4. Line 184: I am not sure whether the expression “lonely people” is appropriate; the sense of loneliness in this study was just a measurement, and some readers might think this expression could induce a form of discrimination.

Answer 4. Thanks for the suggestion. We have rephrased it as follows.

[P.10 L.198] However, among those who reported feeling lonely, higher instrumental support (both receipt and provision) was associated with an *increased* prevalence of hypertension.

[P.14 L.259] In addition, less heavy drinking habit was observed for those reporting loneliness (6.1% vs. 10.5% drank every day heavily (50 g or more pure alcohol)).

[P.16 L.322] Previous literature has documented that the amount of received support may reflect poor health status especially in cross-sectional settings [46,47]. This has been called the support mobilization hypothesis [48], which posits that received support is an indicator of poor health. However, in this study, those who reported feeling lonely were less likely to drink alcohol or to smoke, even though they reported fewer supports. Those who received more instrumental support tended to drink less and smoke, while those reporting more friends tended to be younger, had higher education/income, smoked more, and were more physically active. These patterns tend to suggest that healthier people received more support (see Appendix).

5. Line 185–: Importantly, we cannot interpret a mere statistical interaction as a biological interaction (a mechanism). How about calculating superadditivity, referring to Chapter 5 in Modern Epidemiology 3rd Edition?

Answer 5. Thank you for this important suggestion. We have calculated the relative excess risk due to interaction (RERI) and certificated that there were significant additive interactions between loneliness and each type of social supports. The following sentences were added to the Methods and Results sections. We also cited two articles.

[P.6 L.154] Relative excess risk due to interaction (RERI) was also calculated using estimates from the Poisson regression to investigate whether additive interaction is positive or negative [30,31].

[P.10 L.204] RERI for the interaction between loneliness and both receiving and providing instrumental support was 0.67 (95% CI 0.39 – 0.95, *p* < 0.001) in Model 2, while that for interaction between loneliness and emotional support was 0.17 (95% CI 0.01 – 0.34) for 1 – 5 friends, 0.32 (95% CI -0.14 – 0.78) for 6 or more friends, respectively. That is, the direction of results for additive interaction were consistent with those from the multiplicative interaction.

*References*

[30] Lash TL, Greenland S, Rothman K. Modern Epidemiology, 3rd Edition. 2007 [cited 18 Oct 2021]. Available: <https://www.rti.org/publication/modern-epidemiology-3rd-edition>

[31] VanderWeele TJ, Knol MJ. A Tutorial on Interaction. Epidemiologic Methods. 2014;3: 33–72. doi:10.1515/em-2013-0005

6. Table 2: I would present the estimates and confidence intervals only for the independent variable and interaction terms. You should not present irrelevant rows, all the more so since you did not try to find risk factors.

Answer 6. Thank you for the suggestion. We have removed estimates for covariates from the Tables.

7. Figure 1: The subtitles of the right two graphs should be different. I would also add confidence intervals for each point.

Answer 7. Thank you for your careful review of our manuscript. We have revised the subtitles as per this suggestion.

As for including confidence intervals, we fear that the figures would look messy and detract from our presentation. Also, the figures show the results of model predictions based on the estimates obtained in the regression model. Given that confidence intervals obtained in the prediction procedures were not the same as those presented in the Table 2, we are afraid that displaying the confidence intervals in the figure will confuse readers.

We have updated Figure 1 as follows:

8. Line 198–: I am wondering what the expression “the observed associations were all unchanged and more obvious” means. Could you be more specific?

Answer 8. Following the suggestion, we have added the explanation.

[P.13 L.221] When we excluded covariates from the analyses, the observed associations were statistically significant (i.e., loneliness was associated with hypertension, and there were significant interactions between loneliness and social supports on hypertension) while the magnitude of the estimates became larger; for example, the association between loneliness and hypertension was stronger (PR = 1.33, 95% CI 1.18 – 1.51 in Model 1).

**Discussion**

1. Line 205–: I think you should mention that this study was cross-sectional and the direction of the statistical interaction in the summary paragraph. Furthermore, I would replace the expression “individuals who reported feeling lonely showed a higher prevalence” because prevalence is defined for population, not for a person.

Answer 1. We appreciate the suggestion. As per this suggestion, the following sentence was revised:

[P.13 L.240] In a cross-sectional sample of 763 rural community dwellers in Fujian Province, China, individuals who reported feeling lonely were more likely to have hypertension compared to those who did not feel lonely, especially among older people.

2. Line 215: I do not think the odds ratio of the previous study is necessary unless the figure has a meaning.

Answer 2. We removed the effect size following the reviewer’s suggestion.

3. Line 219: I think you should make it clear what the social threat is.

Answer 3. Thank you for pointing this out. We have amended the sentence as follows:

[P.14 L.252] Hawkley and Cacioppo [3] have argued that loneliness is equivalent to feeling unsafe and those who feel lonely have higher sensitivity to social threat in the environment; lonely individuals see the social world as a more threatening place, expect more negative social interactions, and remember more negative social information.

4. Line 222–: You cannot judge whether people who felt lonely were more likely to be engaged in some healthy behaviors because those associations were subject to confounding. There is a concern for multiple-comparisons, too.

Answer 4. Thank you for raising the important issue. We were trying to illustrate the characteristics of those who showed higher loneliness and those who did not. We have removed the result from regression models and mentioned only numbers (e.g., percentage of having each characteristic) from Table 1.

[P.14 L.257] In our study sample, those who felt lonely reported lower physical activity (38.0% vs. 27.6%), while physical activity was not clearly associated with hypertension in this study. In addition, less heavy drinking was observed for those reporting loneliness (6.1% vs. 10.5% engaged in daily heavy drinking, defined as 50g or more pure alcohol)).

5. Line 222–: The sentence “In our rural setting, drinking and smoking” seems to me subjective. Could you present some evidence to this claim?

Answer 5. Thank you for the question. We have introduced a previous paper as follows:

[P.15 L.270] A previous study that used data from 29 districts of 3 cities in China found that high membership rate in social organizations was associated with higher prevalence of harmful drinking among both men and women. The authors concluded that the Chinese drinking culture may influence drinking behaviors [34].

*Reference*

[34] Nie X, Zhu Y, Fu H, Dai J, Gao J. The “Dark Side” Effects of Social Capital on Harmful Drinking among Chinese Community Residents: A Multilevel Study. International Journal of Environmental Research and Public Health. 2018;15: 2249. doi:10.3390/ijerph15102249

6. Line 235–: I am wondering whether you could check the variable for stress; otherwise, it is not clear what this paragraph was aimed for.

Answer 6. Since we had data of K6 distress scale among those aged 70 years or younger, we have shown the K6 score for those who reported loneliness versus not.

[P.15 L.277] In this study sample, loneliness was significantly associated with stress measured by the Kessler Psychological Distress Scale (K6) [36] among those aged 70 years or younger, which was not investigated among older people since there was difficulty understanding Mandarin Chinese (older people in this area usually communicate in dialect) (mean [SD] 3.7 [3.8] vs. 6.7 [5.4]).

7. Line 249–: Could you please explain what the gift-giving culture? Do you have some information on this feature in your sample?

Answer 7. Thank you for pointing this out. We have added an explanation about Chinese gift-giving culture. Unfortunately, we did not ask about gift exchange in our survey.

[P.15 L.291] Chinese culture (along with other East Asian societies) maintains a tradition of gift exchange for lubricating social relationships with others. Gifts are exchanged between family and friends, but also between co-workers and business associates on significant occasions, such as the Chinese New Year or the mid-autumn festival. In rural areas, gift exchange within social networks functions as a form of informal insurance, creating an obligation on the part of the recipient to reciprocate a favor in the future [41]. For individuals who are feeling lonely, customs surrounding gift exchanges can be a form of social stress [42].

*Reference*

[41] Chen X. Gift-Giving and Network Structure in Rural China: Utilizing Long-Term Spontaneous Gift Records. PLOS ONE. 2014;9: e102104. doi:10.1371/journal.pone.0102104

8. Line 250–: This paragraph should be placed on the Limitation subsection. Furthermore, the expression “inverse association” does not make sense; an association can be bidirectional contrary to a causal relationship.

Answer 8. Following the reviewer’s suggestion, we have moved the sentences to limitation and amended the sentences as follows:

[P.16 L.322] Previous literature has documented that the amount of received support may reflect poor health status especially in cross-sectional settings [46,47]. This has been called the support mobilization hypothesis [48], which posits that received support is an indicator of poor health. However, in this study, those who reported feeling lonely were less likely to drink alcohol or to smoke, even though they reported fewer supports. Those who received more instrumental support tended to drink less and smoke, while those reporting more friends tended to be younger, had higher education/income, smoked more, and were more physically active. These patterns tend to suggest that healthier people receive more support (see Appendix).

9. Line 261– & Limitation: It is important to keep in mind that this is a cross-sectional study, although a longitudinal study does not solve all the issues; how could you tell social relationships were not a confounder but a mediator? If they were just a confounder, the results were not incompatible with the buffering hypothesis at all. Rather, the results without the adjustment for social relationships might be confounded. How could you justify to claim the association was “pronounced” instead of “less biased” under the possibility of confounding?

Answer 9. Thank you for raising this fundamental issue. As you point out, the association between loneliness and social support is likely to be bidirectional, i.e., feelings of loneliness may affect exchange of social support, and exchange of social support may influence feelings of loneliness. Hence, exchange of social support can be simultaneously a mediator and confounder of the association between loneliness and hypertension. The bidirectionality cannot be teased out in cross-sectional data. (It would be also challenging to tease out in longitudinal data, without multiple waves of data capturing changes in loneliness and social support).

We have mentioned this issue in the Limitation section.

[P.17 L.329] We also note that loneliness could have been the product of social support, i.e., those receiving social support tended to feel less lonely. While we assumed temporal ordering from loneliness 🡪 social support, the association between loneliness and social support is likely to be bidirectional. Hence, exchange of social support can be simultaneously a mediator and confounder of the association between loneliness and hypertension. The bidirectionality cannot be teased out in cross-sectional data. It would be also challenging to tease out in longitudinal data, without multiple waves of data capturing changes in loneliness and social support.

**Others**

1. I have the impression that this manuscript needs a revision by a native English proofreader, although I do not feel qualified to judge English as it is not my mother language. Hence, I might fail to get the gist of this manuscript, especially for Discussion.

Answer 1. We appreciate the reviewer for this comment. The manuscript has been carefully reviewed by a native English speaker.

2. Conclusion should be concise; new Discussion cannot be included there.

Answer 2. We appreciate the suggestion. We have removed the following sentences from the Conclusion.

[P.22, Removed] Socially-isolated individuals are at increased risk for feeling lonely, but loneliness is not synonymous with being socially-isolated [5]. Although social isolation and loneliness are usually captured as a similar concept [7], given that result of this study indicated that there was a moderation effect between loneliness (i.e., perception of own social isolation) and social support (i.e., actual substantial/material exchanges with people), it might be important to separately assess them.

I hope you can make use of the primary data in a clear and transparent way and contribute to the literature on social relationships and health.

Answer. Thank you again for your precious time and feedback for the manuscript.

**Reviewer #2**:

The authors examined associations of interaction between loneliness and social support with prevalent hypertension and found paradoxical associations: individuals with both loneliness and high social support were more likely to have hypertension than those with either loneliness or low social support. Those findings may be a fact but look like an artifact. I have several concerns below.

Answer. Thank you very much for your insightful suggestions. Please see our detailed responses to your comments on our manuscript.

1. According to table 1, participants aged 65 years or older look substantially different from those aged 64 years or younger. Since the late '90s, Chinese economic growth became great, and intranational migration from rural areas to industrial or urban areas drastically increased as the authors mentioned in the introduction. This historical background may cause selection bias and result in paradoxical associations. To avoid such historical selection bias, authors should show the results stratified by age as well.

Answer 1. Thank you very much for this comment. Following this comment, we checked if there was interaction between age category (younger than 45 years old, 45 to 64 years, and 65 years or older) and loneliness, but we did not find any evidence of significant interactions (*p* for interaction = 0.77 for those aged 45 to 64 years, 0.52 for those aged 65 years or older, respectively). Thus, we have kept the analysis without age stratification.

We also appreciate the reviewer’s concern as regards selection bias. For example, it is possible that younger people who were healthier compared to others left the area to seek migratory work (healthy migrant hypothesis) and were not included in our survey. We mentioned this issue in the Limitation section.

[P.7 L.163] Fourth, the age-stratified analysis (younger than 45 years old, 45 to 64 years, and 65 years or older) were conducted.

[P.13 L.227] When we conducted the analyses stratified by age group, the significant association was only observed among those aged 65 years or older (PR = 1.26, 95% CI 1.06, 1.51, n = 269), while there was no significant association among younger people (PR = 0.81, 95%CI 0.44, 1.49 among those aged 18 - 44 years (n = 105); PR = 1.02, 95% CI 0.81-1.27 among those aged 45 – 64 years (n = 389)). The interactions between social support and loneliness were also significant among those aged 65 years or older for both instrumental support (loneliness x provision only: PR = 2.05, 95% CI 1.14, 3.71, loneliness x both receipt and provision: PR = 1.47, 95% CI 1.06, 2.06) and emotional support (loneliness x middle support: 1.59 95%CI 1.15, 2.19, loneliness x high support: PR = 1.31, 95% CI 0.99, 1.74).

[P.13 L.240] In a cross-sectional sample of 763 rural community dwellers in Fujian Province, China, individuals who reported feeling lonely were more likely to have hypertension than those who did not feel lonely, especially among older people.

[P.17 L.337] Especially, it is possible that younger, healthier people left the areas to seek migratory work (healthy migrant hypothesis) and were not included in our survey.

2. Alcohol intake is assessed only as frequency, not as the amount. Also, the information about quitting alcohol intake was not available. The dose-response association between alcohol intake and incident hypertension has been well-established. However, such an association for prevalent hypertension did not find in the present study. This discrepancy may happen because quitting or reducing alcohol intake was more likely to occur among hypertensive patients and because alcohol intake was misclassified due to the lack of data on the amount of alcohol intake. Furthermore, the present paradoxical findings may be explained by the binge drinking, not assessed in the present study, among individuals with loneliness and high social support.

Answer 2. Thank you for raising this important point. We have data to estimate the amount of alcohol intake. Results showed that heavy drinking habit was not common among those who were lonely (6.1% among those who were lonely vs. 10.5% among those who were not), and it was not associated with hypertension in this study sample (PR = 0.82, 95% CI 0.63, 1.08). Still, we have decided to include a variable for alcohol consumption with 5 categories (does not drink; 1 or 2 days a week; 3 to 6 days a week; every day (<50g pure alcohol); every day (≥50g pure alcohol)).

[P.6 L.131] Lifestyle factors included alcohol consumption (does not drink; 1 or 2 days a week; 3 to 6 days a week; every day (<50g pure alcohol); every day (≥50g pure alcohol)), smoking (never smoked; stopped smoking; currently smoke), and physical activity. The amount of alcohol was calculated if the participant answered that they consume alcoholic beverages every day. For the usual daily quantity consumed, participants were asked to report types (beer (~4%), rice wine (~25%), strong spirits (~50%), wine (~10%)) and quantity (bottle for beer, which is usually 640 ml in China, and liang (Chinese ounce equivalent with 50g) or for others). Pure alcohol consumption was then calculated and those who consumed 50g or more pure alcohol daily were defined as having heavy drinking habit [27].

[P.14 L.255] Social threat can cause diminished self-regulation, so that people with loneliness tend to have worse health behaviors such as more alcohol consumption and less physical activity [27,28]. In our study sample, those who felt lonely reported lower physical activity (38.0% vs. 27.6%), while physical activity was not clearly associated with hypertension in this study. In addition, less heavy drinking was observed for those reporting loneliness (6.1% vs. 10.5% drank every day heavily (50 g or more pure alcohol)).

3. In table 3, women were more likely to have hypertension than men. Is this true? In general, men are more likely to have hypertension than women. The authors need to confirm the reference category of sex in table 3. If no error, there may be selection bias between men and women.

Answer 3. We have double-checked the data and confirmed that women were more likely to have hypertension than men in our study sample. We agree with the reviewer that this was an unexpected result. While this is a chance finding, we also found other studies that showed higher prevalence of hypertension among women compared to men. For example, a study conducted in southwestern China showed a higher prevalence of hypertension among women vs. men after the age of 64 while the opposite trend was observed in younger age groups [1]. The same trend was observed for the representative population in the US [2] and Korea [3].

Given that the gender difference in hypertension was not our primary focus in this study, we decided not to include this discussion in the manuscript. If the editor prefers us to include, we are happy to amend the manuscript further.

*References*

1. Huang, XB et al*.* Prevalence, awareness, treatment, and control of hypertension in southwestern China. Sci Rep 9**,** 19098 (2019). https://doi-org.ezp-prod1.hul.harvard.edu/10.1038/s41598-019-55438-7
2. Go AS et al; American Heart Association Statistics Committee and Stroke Statistics Subcommittee. Heart disease and stroke statistics--2013 update: a report from the American Heart Association. Circulation. 2013 Jan 1;127(1):e6-e245. doi: 10.1161/CIR.0b013e31828124ad. Epub 2012 Dec 12. Erratum in: Circulation. 2013 Jan 1;127(1):doi:10.1161/CIR.0b013e31828124ad. Erratum in: Circulation. 2013 Jun 11;127(23):e841. PMID: 23239837; PMCID: PMC5408511.
3. Choi HM et al. Sex differences in hypertension prevalence and control: Analysis of the 2010-2014 Korea National Health and Nutrition Examination Survey. PLoS One. 2017 May 25;12(5):e0178334. doi: 10.1371/journal.pone.0178334. PMID: 28542557; PMCID: PMC5444798.

4. In table 3, age is better adjusted for as fifths or other categories than the continuous value because the association between age and hypertension can be non-linear rather than linear. In addition, for BMI, the reference category should have a narrower range such as 18.5-22.9 kg/m2. The overweight criterion for Asians is 23.0 kg/m2.

Answer 4. We appreciate the suggestion. We have added both age and age-squared to account for the non-linear association between age and blood pressure. We have changed the cut-offs for BMI and adopted that for Asians (underweight: <18.5; normal weight: 18.5 – 22.9; overweight: 23 – 27.4; obese: ≥27.5). We did not see any material changes in the results following these changes.

[P.5 L.124] Demographic and socio-economic data included age (in years), age-squared, sex (male; female), body mass index (kg/m^2^) which was categorized into four levels according to Asian cut-off values (underweight: <18.5; normal weight: 18.5 – 22.9; overweight: 23.0 – 27.5; obese: ≥27.5) [26],

[P.6 L.152] Covariates included age, age-squared, sex, body mass index, marital status, educational attainment, employment, household income, alcohol consumption, smoking, and physical activity.
